# A Feasibility Study Describing the Successes and Challenges of Implementing a Virtual Community Health Worker Training Among High School Students Participating in a Summer STEM Enrichment Program

**DOI:** 10.15695/jstem/v7i1.01

**Published:** 2024-01-22

**Authors:** Archana Bhavani Vasanth Kumar, Gia Grier McGinnis, Laundette Jones, Erin R. Hager, Sequoia L. Wright, Cara Felter, Greg Carey, Bret Hassel, Arletha W. Livingston, Elizabeth A. Parker

**Affiliations:** 1 Departments of Epidemiology and Public Health, University of Maryland School of Medicine, Baltimore, MD;; 2 Departments of Center for Community, Service, and Justice, Loyola University Maryland, Baltimore, MD;; 3 Departments of University of Maryland Greenebaum Comprehensive Cancer Center, Baltimore, MD;; 4 Department of Population, Family and Reproductive Health, Johns Hopkins Bloomberg School of Public Health, Baltimore, MD;; 5 Departments of Pediatrics, University of Maryland School of Medicine, Baltimore, MD;; 6 Departments of Physical Therapy and Rehabilitation Science, University of Maryland School of Medicine, Baltimore, MD;; 7 Departments of Microbiology and Immunology, University of Maryland School of Medicine, Baltimore, MD;; 8 Departments of Morehouse School of Medicine, Atlanta, GA

**Keywords:** Community Health, STEM Enrichment, Virtual Programming, Out-of-school-time STEM Initiatives, UMB CURE

## Abstract

University of Maryland, Baltimore CURE Connections (UMB CURE) connects West Baltimore high school students with STEM enrichment including hands-on research and community outreach. This study’s purpose was to describe successes and challenges of implementing the virtual Community Health Worker curriculum during the summer programming for UMB CURE high school scholars. This certificate-based program was designed to teach students about the community health field while providing training that demonstrates competence as a community health worker. The training was implemented over two summer sessions (2020 and 2021). Scholars completed a survey to assess program satisfaction. A subset of scholars completed qualitative interviews that focused on scholars’ summer program experience and recommendations for program improvement. Engagement metrics (scholar participation, retention) were compiled. Overall themes from qualitative interviews included (1) overall summer program experience, (2) about the Morehouse curriculum, (3) advice for future scholars, (4) in-person versus virtual summer program, and (5) recommendations for the program. While the program was generally well-received, scholars required more instruction and guidance than anticipated. Many found the required assignments challenging to navigate, citing virtual instruction as a reason. Scholars also requested more hands-on synchronous STEM-focused activities. These data will be used to modify future programming to engage scholars in out-of-school-time STEM initiatives.

## INTRODUCTION

Health status is unequal among all population groups leading to health inequities, which are both undesirable and unfair, yet avoidable (Penman-Aguilar et al., 2016). The communities profoundly affected by the health inequities are recognized as underserved or vulnerable populations (MUA Find, 2020). Health inequities are the differences in the distribution of health services and resources between different populations that stem from the people’s social conditions (Lucyk and McLaren, 2017; Penman-Aguilar et al., 2016; World Health Organization, 2008). This concept of health inequity takes root from the social determinants of health, and the social conditions in which people live and grow (Benach et al., 2010; Braveman and Gottlieb, 2014; Spruce, 2019). In order to reduce health inequities, it is imperative to focus on strategies that address the social determinants of health (Penman-Aguilar et al., 2016). Targeting upstream social determinants, including access to enhanced early childhood education and employment interventions, results in better health outcomes among populations facing health inequities (Thornton et al., 2016). The role of a community health worker (CHW) is to bridge access and resource gaps between underserved communities and facilitate equitable access to health resources and social services (Olaniran et al., 2017).

A CHW is a member of the community they intend to serve (Olaniran et al., 2017). CHWs are paid workers or volunteers who work in liaison with the local health care system by providing education, outreach and resources to community members in order to promote access to healthcare (Gadsden et al., 2021). The primary intention for developing these outreach workers into a workforce is to break the barriers to complete utilization of healthcare facilities caused by non-medical social situations (McCray et al., 2020). In the U.S., CHW training programs are predominantly designed for adults. Recently youth engagement in CHW programs, health leadership, participatory research, and social projects has increased (El-Awaisi et al., 2016; Zheng et al., 2021). The increased popularity of both in-school and after-school program settings has harnessed youths’ potential to benefit their community through empowerment (To et al., 2021). The concept of youth participation has yielded organizational sustainability and effectiveness in social and economic development (Hull et al., 2018; O’Donoghue et al., 2002) and community research (Santilli et al., 2011).

To create CHW opportunities for youth, the Morehouse School of Medicine (MSM), in collaboration with the American Cancer Society Southeast Region and the Georgia Department of Public Health, developed a CHW training program curriculum (Williams-Livingston et al., 2020) for high school students and young adults (The High School and Young Adults Community Health Worker training program| Morehouse School of Medicine, 2021). The MSM has been training CHWs for more than 15 years. However, it implemented the CHW training program for high school students only in 2016 as a pilot program (Williams-Livingston et al., 2020), which their team then transitioned to the virtual platform and the first Train-the-Trainer was made available in February 2019. The High School and Young Adults Community Health Worker (HSYACHW) training program is the first of its kind in the U.S. (Morehouse School of Medicine, 2016). The MSM HSYACHW summer training program is a seven-week training program that includes shadowing experience, and self-guided and interactive sessions meant to be implemented virtually for those outside of the MSM catchment area.

The University of Maryland, Baltimore Continuing Umbrella of Research Experiences (UMB CURE) was developed in 2015 as a joint initiative by the UMB’s President’s Office and the University of Maryland Marlene and Stewart Greenebaum Comprehensive Cancer Center. It started as a middle school pilot program funded within the National Institutes of Health’s (NIH) National Cancer Institute’s national CURE program. Additional funding from NIH’s National Institute of General Medical Sciences, through its Science Education Award Program, allowed the program to expand to high school as the scholars graduated from middle school. The program’s primary focus is to expose West Baltimore youth to diverse career and educational pathways in healthcare, research, STEM (science, technology, engineering, mathematics) and higher education. UMB CURE is comprised of middle school and high school components and is part of a growing national effort to diversify the STEM and healthcare workforce and reduce disparities. The high school component, UMB CURE Connections (C2), is comprised of STEM Saturdays (held throughout the school year) and two six-week summer sessions; both elements of the curriculum are supported by robust near-peer and mentoring by students from the seven UMB professional schools. Due to social and physical distancing requirements resulting from the COVID-19 pandemic, C2 abruptly transitioned from in-person programming to a virtual platform in 2020. To accommodate the abrupt change in programming, scholars participated in the virtual HSYACHW starting in the summer of 2020. This study describes challenges and successes of implementing the MSM HSYACHW training program curriculum during the summer of 2020 (year 1) using dimensions from the RE-AIM (reach, effectiveness, adoption, implementation, maintenance) framework (Kessler et al., 2013; Kwan et al., 2019) to evaluate the implementation of the virtual training. We also describe program modifications made by UMB CURE in 2021 (year 2) based on scholar feedback from year 1 and compared feedback from scholars who participated in the year 1 and year 2 program.

## METHODS

### Program Description: UMB CURE Connections (C2) Summer Program.

UMB CURE Connections (C2) is an integral component of a minority STEM education pipeline, connecting West Baltimore high school students with STEM enrichment including hands-on research and community outreach to a network of minority-focused college programs at UMB and its partner institutions. During the summer program, scholars participate in an immersive STEM curriculum consisting of various components to engage them productively and improve their interests towards a STEM career in the future. Through a partnership with the Baltimore City Mayor’s Office YouthWorks program, scholars are provided workforce development opportunities and payment compensation and attend daily virtual sessions for 6 weeks (20 hours/week).

In response to the sudden transition from in-person programming to the virtual platform as a result of physical and social distancing requirements put forth from the COVID-19 pandemic in 2020, the C2 summer program implemented the existing MSM HSYACHW virtual training program for all high school students (rising 9th-11th) participating in the summer program (total n=45). This program was originally developed by MSM as in person program, and adapted by MSM to be virtually implemented in other settings (The High School and Young Adults Commuity Health Worker training program|Morehouse School of Medicine, 2021). Students were split into groups by grade; each group was led by an instructor (2 C2 program coordinators; 1 public school teacher) who completed the MSM train the trainer orientation. The CHW training program was originally developed to train students in the community health field through the completion of 18 online modules available via the Canvas platform (The High School and Young Adults Community Health Worker training program|Morehouse School of Medicine, 2021). Each of these modules are integrated with STEM-related learning materials consisting of videos, articles, and career panel interview opportunities. The modules are structured to provide an immersive learning experience for the scholars, with numerous assignments to train them to be successful CHWs. The scholars are required to submit assignments in the form of written answers, VoiceThread (comment through voice recording within a video) assignments, and Prezi (interactive presentations). The CHW training program was the primary component of the summer program in 2020 and scholars were instructed to complete each module over the 6-week program. The modules were expected to be completed asynchronously, with a 30-minute live check-in period with instructors at the beginning and end of each day. Upon completion of all 18 modules, participants received a certificate demonstrating competence as a CHW. The CHW training program was supplemented with live guest lecture sessions for all scholars. A subset of students also participated in a week of forensics science focused curriculum (rising 10th graders, n=16), or a week of leadership focused curriculum (rising 9th graders, n=12).

As a strategy to encourage participation in programming and ensure student participation was not hindered by technological barriers, staff called families to survey their available resources and identify scholars who lacked the at-home technology (no smart phone, tablet, or computer) to perform virtual tasks. Existing CURE Chromebooks were provided to a small number of families who needed equipment. At the time of programming, a national internet provider was offering a free trial of their Internet Essentials package for certain households in response to COVID-19. CURE families were referred to that service if they did not currently have a home-based internet provider.

### **Program Evaluation and RE-AIM Dimensions**.

RE-AIM framework, which was introduced by Glasgow et al. in 1999 is the most popular framework used in public health for planning and evaluation of programs (Holtrop et al., 2021). Dimensions from the RE-AIM framework (Kessler et al., 2013; Kwan et al., 2019) were used to evaluate program implementation. This RE-AIM framework model, proposed to evaluate public health interventions, serves the purpose through five dimensions. These five dimensions that RE-AIM stands for and assessed are Reach, Efficacy, Adoption, Implementation, and Maintenance (Glasgow et al., 1999). Addressing these five dimensions of a program’s outcomes will eventually help in evaluating the program impact and its long term sustainability (Kwan et al., 2019). To assess ‘Reach,’ we included the percentage of scholars who participated in the summer program. For ‘Effectiveness’ and ‘Implementation,’ a team member reviewed the Canvas site to collect metrics related to assignment completion, time spent on various modules and overall module completion. If they completed all 18 modules, scholars received the CHW certificate. The number of scholars who received the certificate was also recorded.

Following the summer 2020 program, scholars completed a brief survey to assess overall program satisfaction and provide open-ended feedback about the program. Scholars were asked the question on a Likert scale, “On a scale of 1–10 (1 worst, 10 best) how would you rate your experience in C2 summer?” The open-ended questions were, “What did you like the most about C2 summer programming?” and “What did you like the least about C2 summer programming?” Adaptations to the protocol between 2020 and 2021 accounted for feedback survey data as well as informal verbal feedback from students to the instructors during synchronous summer programming check-in sessions about module preferences and acceptability. Modifications to the protocol between 2020 and 2021 are documented in Table 2.

At the end of the summer program 2021, scholars from both 2020 and 2021 programs were interviewed using semi-structured guided interviews to understand scholars’ perceptions of the program to understand effectiveness and adoption of the intervention. We obtained IRB approval through the UMSOM as well as written parental consent for the scholars who volunteered for the qualitative interview. In addition, a scholar assent form was completed for each scholar under the age of 18. The participating scholars attended a one-on-one semi-structured interview. The interview process lasted for approximately 15–20 minutes.

We employed convenience and quota sampling procedures to select the participants. The interview time was set to accommodate the scholars’ convenience given that their regular school had started. The interview commenced after receiving verbal consent/assent from the scholars. The interview was conducted using the Zoom video conferencing software and recorded for transcription purposes.

The interview questions addressed the scholars’ experience and feedback pertaining to the overall summer program, MSM modules, and their recommendations to improve the program for future scholars. This interview utilized open-ended questions.

After the interview process, transcription was completed, following which the recording was deleted. Deductive open coding was done for all six transcriptions, then reanalyzed for more focused coding to collapse and combine open codes from the six interviews. Themes were developed based on the generated codes. Given the small sample size, this process was conducted by a single individual.

## RESULTS

The first cohort from 2020 (year 1), who participated in the MSM HSYACHW training program, consisted of 45 scholars from rising ninth, tenth and eleventh grades (66% of eligible scholars). The 2021 (year 2) cohort consisted of 7 (78% of eligible scholars) scholars. Summer programming is not mandatory for participation in the UMB CURE Scholars Program. The top reasons for declined participation included the virtual nature of the program after having spent the prior semester in virtual school, and/or seeking employment opportunities that allowed the scholars to be in-person. Available participant demographics and quantitative data from the Canvas website are presented in Table 1. Overall, for the two years, there were 52 participants, consisting of 32 males and 20 females. Scholars’ mean age was 16 years. On average, scholars completed 28.21 assignments out of 75; however, there was considerable variation with a few scholars not completing any assignments to some scholars completing as many as 74 assignments. The average time spent on the Canvas app was 33.37 hours; however, time spent on the Canvas app did not correspond to the number of assignments completed. The CHW certification was received by three scholars, all of whom belonged to the 2020 cohort and were rising 9th graders (one male, two females). None of the scholars from the 2021 cohort received the certificate.

### 2020 Feedback Survey Results and Subsequent 2021 Summer Modifications.

Based on scholars’ feedback, the MSM HSYACHW training was modified in 2021 to exclude specific modules in the curriculum, and scholars were only required to complete 12 of the 18 modules (Table 2). Several modules were removed based on the informal scholar feedback from 2020 as well as the C2 program coordinator’s knowledge of where the rising 9th graders in the summer 2021 cohort were academically. For example, the CITI and HIPAA training were too much for the rising 9th graders the previous year and thus were removed in the second year. Decreasing the number of modules covered during summer programming allowed us to provide additional time for scholars to work synchronously with an instructor to complete the modules. However, after completing the program, all modules were accessible to the scholars for those interested in completing the training program and receiving the CHW certificate on their own time. Because the older students had completed this training the prior summer, the 2021 cohort only included rising 9th graders. The 2021 cohort was led by an MPH student serving as a summer program assistant who also completed the MSM train the trainer orientation. This assistant was supervised by one of the C2 program coordinators who led the course in 2020.

The 2021 summer program incorporated different workshops, namely Scratch videogame coding, Genomics and Radiology workshops. Two weeks of the program comprised of video game coding workshop that utilized the SCRATCH programming, which provides an introduction to coding by teaching students how to create digital stories through coding. As a final project, they chose a public health-related topic and created a story to advocate for the chosen topic. The genomics workshop delivered by the Personal Genome Diagnostics (PGDx) team provided the scholars with an overview on how genome sequencing can be used in immunotherapy for the benefit of cancer treatment. The scholars followed a case study using the tools learned about central dogma and gene expression to determine the course of treatment for a lung cancer patient. During the radiology workshop conducted by the University of Maryland School of Medicine (UMSOM) Radiology Department, scholars learned about the different imaging techniques and technologies, including the various planes and modes for imaging and classified images of X-rays, CT, and MRI scans. The faculty from the department also provided a brief information session about career pathways in the radiology field.

In response to feedback from scholars participating in the 2020 Summer program about additional interaction with peers and instructors, we adapted the 2021 Summer program to incorporate more synchronous virtual led activities, including live weekly guest lectures with speakers from various STEM careers. Scholars also received lab kits aimed to boost their STEM skills and complemented the content presented in the CHW modules. We incorporated sessions where the instructor led the scholars in completion of these lab kits. The kits provided include the lung volume kit, sugar metabolism kit, chemistry of food experiment kit, and the hydraulic robotic arm. The students were excited while working on building the hydraulic arm. Students tested their final output and students shared their engineering skills by operating the robotic arm while on google meet. Apart from stimulating their STEM skills these hands-on activities also improved their focus and commitment toward completing a project and keeping the students engrossed. The students were also provided with a body weight scale, a measuring tape, and a blood pressure monitor that helped them to practice collecting health-related data such as vital signs on family members and friends. This reinforced the content learned within the modules focused on public health. Having these hands-on experiences encouraged students to be more involved and engaged in the program.

### Semi-Structured Guided Interviews.

More than 10 scholars, who had signed initial consent/assent, were invited to participate in the interviews of which six responded and agreed to participate. Of those interviewed, four scholars were from the 2020 summer program and two were from the 2021 summer program.

Themes that evolved from the final codes best summarized the interview data. These themes developed as a result of the common codes from all six interviews. These themes include (1) overall summer program experience, (2) about the MSM curriculum, (3) advice for future scholars, (4) in-person versus virtual summer program, and (5) recommendations for the program. Some themes were further classified into sub-themes, and the themes represent the questions pertaining to the program experience within the interview guide. All key themes, and excerpts from the interview that serve as exemplars for the themes are presented in Table 3. The interview guide is presented in the Appendix.

1. **Overall summer program experience**. The scholars had mostly positive feedback about their overall CURE summer program experience. All scholars felt they learned new things and most had fun in the process, which proved to be an encouraging response. The codes “fun” and “learning” emerged in multiple interviews. Scholars mostly had a brief straightforward response when answering this question. A scholar appreciated how the program incorporated different interesting STEM components. The opinion of fun learning experience remained unchanged with the scholars from both 2020 and 2021 summer program.
  - **Positive feedback.** Responses regarding what they liked about the summer program varied between scholars. One scholar enjoyed the interactive sessions, while a second scholar liked the fact about not having to sit in front of the camera all day long, and instead work on the modules by themselves. The hands-on activities implemented for the scholars during the 2021 program, based on the feedback survey from the previous year, was well appreciated by the scholars.
  - **Negative feedback.** While the scholars had different aspects that they disliked most opinions were in consensus to the program being held in virtual setting, which was inevitable due to the COVID-19 pandemic. The obstacles surrounding the virtual learning include but are not restricted to issues with communication and finding a place in their home where they would not be disturbed. Especially for those scholars with younger siblings, many reported getting distracted from their work. The pandemic situation worsened everything for school going children, as the schools had to resort to fully virtual classes. Having to attend classes online for an entire academic year made it tiring for the scholars to continue the CURE summer program online as well.
2. **About the Morehouse Curriculum.** The scholars had many things to say about the modules and the assignments contained within. The three scholars who completed all 18 modules and received the CHW certificate are from the 2020 summer program and of the three scholars who did not receive the certificate, one belonged to the 2020 cohort and two were from the 2021 cohort. None of the scholars from the 2021 summer program completed the 18 modules. Some scholars offered opinions about the different modes of assignment submission, while some commented on the length of the modules as a whole.
  - **Complexity of assignments**. The scholars were required to complete each module by watching educational videos, reading articles, and responding to questions. The assignments were supposed to be answered in the form of a short essay, Voice Thread (voice recordings within the videos), and Prezi (presentations). While one scholar noted that the assignments took longer and required detailed answers, most found the Voice Thread and Prezi assignments to be challenging to navigate.
  - **Length of the modules.** The scholars shared their opinion that the summer program duration was sufficient to complete the modules; however, all scholars reported that they had to put in extra hours to complete the modules. According to the scholars, the complexity of certain assignments prolonged the time taken to complete the modules. Some modules required reading articles and answering through essays, which ultimately prolonged the time needed to complete the modules. Most scholars shared their opinion about the length of the assignments.
3. **Advice for Future Scholars.** The interviewees were asked what they would advise future scholars to encourage them to receive the CHW certificate. The scholars mentioned communication amongst themselves and with the instructor and being proactive as the keys to successful completion. Two scholars mentioned mentor and peer support as a significant component in accomplishing the task. Of the two scholars who had not completed all the 18 modules to receive the CHW certificate, one completed the required 12 modules for the summer program. However, both scholars were positive about completing all the 18 modules when questioned about how motivated they were in receiving the certificate.
4. **In-Person versus Virtual Program.** The CURE summer program switched to a virtual program for the years 2020 and 2021. Even though the Morehouse CHW training modules are structured to be taken virtually, these modules can be completed during in-person summer program where all the scholars and mentors will be present in the same room and participate actively. The universal response from the scholars on their preference was in-person program when compared to virtual program. All scholars preferred an in-person summer program format, citing reasons such as the presence of mentors, who can help and guide the scholars through the modules, should challenges arise, and being able to interact with peers. Other reasons quoted by scholars in opting for an in-person program were lack of distraction that can be witnessed when working on modules at home, and the being able to tell if scholars are genuinely participating.
5. **Recommendations for the Program.** It was not surprising to find out that all the scholars from the 2020 cohort, without exception, recommended having more hands-on activities and interactive sessions, that can make the program more interesting and engaging. The reason that these recommendations were not surprising comes from the fact that the CURE program had taken up their recommendations from the survey conducted in year 1 and has introduced more hands-on activities for the Year 2 summer program. While these were the recommendations from the scholars from previous year, the scholars from Year 2 who benefitted from these feedbacks, had other recommendations. The Year 2 scholars recommended finding a way to make the hands-on sessions that complement the modules to be more interactive, where all participating scholars update on their progress. They believed that going to the in-person program would be a solution to this lack of communication and this will also reduce the screen time for everyone, which would be an added benefit.

## DISCUSSION

Our study describes the implementation and evaluation of a virtual CHW training program into summer programming for high school students, and highlights challenges faced when pivoting to the virtual setting. Overall while the program was generally well received, scholars required more instruction and guidance than anticipated or planned and many found the required assignments to be challenging to navigate. Present-day CHW programs are centered around rendering services to say communities facing health inequities deprived of health care needs (Hartzler et al., 2018; Lehmann and Sanders, 2007). Engaging youth in CHW training may be a strategy to promote health promotion among communities at highest risk of poor health outcomes, such as those in Baltimore City. The expertise of CHWs in cultural competence has become indispensable to the healthcare workforce (Kash et al., 2007). The need for an intense and tailored CHW training program arose out of the need for CHWs to close the gap in healthcare access among the underserved population. CHW training in the US states happens through different modalities. The three most common training formats include “state certification program,” “community college training,” and “agency-level training.” However, since the early 1980s, the age group of the CHW indicates the possibility of adults ranging from 19 years to 57 years. However, not much literature has explored the age of CHWs. To meet growing expectations and expanding role of CHWs appropriate training supported by continuing education is deemed necessary to guarantee quality outcomes (Adams et al., 2021; Brown et al., 2006; Lehmann and Sanders, 2007; Olaniran et al., 2017).

While empowering students and creating a trusted relationship with their community, community leadership projects also develop their social and emotional well-being (Nabors et al., 2018). Involving community members to bring about changes we anticipate within the community is more effective. Moreover, engaging youth in advocacy programs has two-way benefits for community members and youth health advocates (Millstein and Sallis, 2011). All these benefits can be witnessed from the other youth programs countrywide. The Community Alliance for Research and Engagement (CARE) program in Connecticut aims to improve the underserved and minority population in New Haven (CARE, 2021). The CARE program actively included high school students through an internship program to do community asset mapping, which turned out to be a successful endeavor (Santilli et al., 2011). The Teen Health Leadership Program (THLP), which is still in its evaluation phase, completely engages at-risk students to promote health information through advocacy and outreach (Keselman et al., 2015). The CalFresh initiative, introduced in 2016, changed its approach from serving the youth to engaging them as a part of its program. The program’s focus is to empower children in the age group 12–18 years on nutritional education and physical activity. These children belonging to vulnerable communities will utilize their newfound knowledge and skills to improve their community (Louie et al., 2017). Therefore, strategies to introduce these trainings into high school programs may be beneficial to promote health and community engagement among youth. The availability of a primarily asynchronous, self-paced curriculum, like the MSM program evaluated here, may expand opportunities for more youth to earn a CHW certificate.

As is common when implementing an evidence-based program in a novel setting, adaptations were made. Using the RE-AIM framework, the C2 MSM HSYACHW adaptations were systematically documented in the 2nd year of implementation. The modifications made to the summer program include incorporating hands-on activities that actively engaged the scholars, other than the time they were working on the modules on their own. These hands-on activity kits were chosen to enhance the STEM curricular experience and at the same time to complement the contents presented through the modules. The hands-on lab kits, which are an integral part of STEM experience (Lichtenstein and Phillips, 2021), were welcomed by the new scholars as can be witnessed from their interview responses. Following the scholar feedback gathered at the end of 2020 MSM HSYACHW training program, a modified version of the digital learning curriculum was implemented for the 2021 batch. This modified version espoused more hands-on activity since it was most sought after and highly request by the scholars’ of 2020 cohort. The importance of hands-on activities has been cited throughout literature. Hands-on activities are fun and effectual ways to learn (Roden et al., 2018), by propagating learning by doing. Through hands-on students move on from being passive learners to active participants (Sivan et al., 2000). While these hands-on lab kits were engaging and invigorating, due to the virtual environment each activity consumed more time than previously scheduled.

Secondly, the virtual nature of the program deemed it impossible to identify if all the scholars were actively participating or not. Thus, the duration of the summer program seemed less optimal to complete all the lab activities as pre-planned given its virtual nature. Another crucial challenge with virtual learning from home is the access computer system and internet connection (Morris et al., 2021). While the UMB CURE team provides scholars with Chromebook for their CURE related activities, we cannot deny the problems arising from poor internet connection (Morris et al., 2021).

A limitation with this study is the number of study participants, who may not be representative of the entire scholar population in the program. The number of students significantly varied between the two years. The 2020 summer program consisted of a larger group of students, as it included all scholars from three different grades, while the 2021 summer program consisted of scholars only from one grade to enroll for the CHW training program. Considering the number of scholars in participation, a qualitative interview was conducted to evaluate the program experience in detail. Though many participants and their parents had provided prior assent and consent, approximately 40% did not respond to the interview invitation, leading to a possible non-response bias. However, since the scholars had intersecting responses to the interview questions, this gives a fair insight into the expectations of scholars from the summer program and the Morehouse CHW training modules. Another limitation was identified within the Canvas app where the scholars had to complete their MSM HSYACHW training modules. The time spent on the app did not correspond to the number assignments completed by individual scholars. Thus, a more reliable and effective method should be made available or developed to assess the exact duration of time spent by the scholars on active engagement within the modules. Finally, while we didn’t directly ask scholars about their experience with the Canvas software, some participants did say that the instructional video at the beginning was not particularly helpful for them. Difficulties using the software may have occurred because it was likely the first time that most students used this software; thus, in the future we will include additional training on utilizing the online platform prior to initiating the program. In the future, it would be interesting to compare our sample demographics to other programs utilizing the MSM curriculum and compare metrics of success. Given that the three students in our program who completed the certificate were rising 9th graders, we do not feel that this age is too young for such programming. However, our overall target recruitment area for the UMB CURE program includes multiple neighborhoods across West Baltimore. Baltimore Neighborhood Indicator Alliance data (2018) indicate that the Poppleton/The Terraces/Hollins Market neighborhood has the largest percentage of families living in poverty in Baltimore City (42.8%). In Southwest Baltimore, 31.7% of residents did not graduate HS, and few (6.4%) have a bachelor’s degree. According to the Maryland State Department of Education, the graduation rate in 2022 was 68.65%, and the percent of students in high school who were proficient in math and English language arts were 13.3% and 42%, respectively. These statistics demonstrate the need for programs targeting academic enrichment, and workforce and economic development among youth in Baltimore City.

The CURE team has been amenable to feedback from the scholars in the past, which will serve as the key to the sustainability of the program. The CURE program has once again started its in-person program, and this could overcome the obstacles posed by the virtual program. All the scholars who interviewed recommended more interactive sessions and addressed the one hindrance to a successful completion of the program to be its virtual setting. The CURE program, by focusing on integrating more of this component, combined with the in-person program, may see improvement in the number of scholars committing to the program and who successfully complete the CHW training program. If the CURE program were to continue to offer the CHW training in future summers, it may be valuable to implement the original in-person CHW training program and compare the number of students who opt to receive the completion certificate as compared to the virtual setting.

The scholars enrolled in the CHW training program should be evaluated every year, to make periodic modifications to the program to best benefit the scholars. Holding focus groups will be a good approach for larger groups. On the other hand, conducting semi-structured qualitative interviews with open ended questions can help us discern what the scholars really experience and expect of this program.

Overall, implementing the virtual community health worker training program had both successful components and challenging aspects, which were evident from the student interviews and relatively few students completing the CHW certificate. Despite these challenges, our program continues to utilize feedback from scholars to design STEM based programming that is academically enriching while recognizing scholars need the summer to reset from the school year. We included hands-on activities whenever possible, so they are actively engaged in the learning process versus having to sit in a lecture, read from a textbook, or watch a screen without direct interaction with the speaker or presenter. Continuing to incorporate these recommendations and implementing necessary changes for the subsequent student cohorts is crucial to ensure continued success of this program.

## Supplementary Material

Virtual Community Health Worker Training Program – Vasanth Kumar, et al. Appendix. Interview Guide

## Figures and Tables

**Table 1.** Participant demographics and summary of Canvas metrics (n=52).

**Sex (n)**	
Male	32
Female	20
**Grade level (n)**	
Rising 9th grade (2020 and 2021)	25
Rising 10th grade	15
Rising 11th grade	12
**Average number of assignments completed** (max =75)	28.21
**Average time spent on Canvas portal** (hours)	33.37
**Average program satisfaction**	
“On a scale of 1–10 (1=worst, 10=best) how would you rate your experience in C2 summer?”	8.1/10

**Table 2.** Comparison of required modules and order of completion from Summer 2020 to 2021.

Morehouse Modules: Summer 2020	Morehouse Modules: Summer 2021
Module 1: The role of the CHW in Health Promotion, Introduction to Community Health Work, the Role of the CHW, Qualities of CHWs…	Module 17: Community Health Project
Module 2: The US Health Services System, Population/Community Health, Social Determinants & Barriers to Compliance	Module 1: The role of the CHW in Health Promotion, Introduction to Community Health Work, the Role of the CHW, Qualities of CHWs.
Module 16: Shadowing	Module 2: The US Health Services System, Population/ Community Health, Social Determinants & Barriers to Compliance
Module 3: Bioethics, Privacy, Confidentiality, HIPAA and SBE Research Training	Module 13: Community Assessment, Community Engagement, and Windshield Survey
Module 4: Effective Communication, Interpersonal Communication, and Motivational Interviewing	Module 5: Cultural Competency and Advocacy
Module 5: Cultural Competency and Advocacy	Module 6: Public Health, Health 101, and Immunization
Module 6: Public Health, Health 101, and Immunization	Module 12: Taking Vitals, Case Management, and Motivational Interviewing
Module 7: Beginning Anatomy and My Health	Module 7: Beginning Anatomy and My Health
Module 8: Chronic Disease	Module 8: Chronic Disease
Module 17: Community Health Project	Module 9: Mental Health
Module 9: Mental Health	Module 14: Health and the Environment
Module 10: Sexual Health and Doula	Module 15: Integrative Health Nutrition & Physical Activity
Module 11: Data Science & Data Management	Final Presentation: Community Health Project Due
Module 12: Taking Vitals, Case Management, and Motivational Interviewing	
Module 13: Community Assessment, Community Engagement, and Windshield Survey	
Module 14: Health and the Environment	
Module 15: Integrative Health Nutrition & Physical Activity	
Module 18: Public Speaking and Presentation Skills	
Final Presentation: Community Health Project Due	

**Table 3.** Themes and selected quotes from the qualitative interviews with Scholars.

Themes	Example Quotes
Overall Summer Program Experience	“Umm it was really good… it was a good learning experience. I had a lot of fun.”
	“…it was made up of different things of science like engineering, chemistry, anatomy. So, that was very interesting.”
Positive Feedback	“The thing I like most is when we did like the physical labs……And also, we did the virtual escape rooms”
	“I like the fact that….we would just work on our own……, and if we needed help, we could ask for help.”
Negative Feedback	“…like one thing I didn’t like about the virtual experience was it was really hard to communicate. Like you had to find a quiet place in your room”
	“What I didn’t like, like I said was, us being on a computer, and not in-person”
**About the Morehouse Curriculum**	
Complexity of Assignments	“I didn’t like doing the prezis because I found them hard. I didn’t like finish some of them and I had to finish them in the summer. I had to get help on them cos I didn’t like really understand how to do it. So, I thought that part was hard for me.”
	“…. read some articles, and then make a voice recording so I believe that’s like really the only times when it took a long time to like complete some of the modules”
Length of Modules	“watching the videos took a lot of the time and just like analyzing the whole video”
	“…afterwards it was almost like homework, so like that took longer than like, expected”
Advice for Future Scholars	“Umm don’t necessarily give up just yeah… just ask your teachers for help. Teachers and mentors are there to help you and you can use that extra help”
	“not to let the modules to build up and if you can just like knock a couple out a day…… So that’s really the only suggestion that I have is to kind of be proactive”
In-Person Vs. Virtual Program	“So, some directions on the modules were confusing… I think it might be better to get a real-life example from another student in-person”
	“when you’re sitting at home you can get distracted easily and just like walk away from the computer and just not come back…… If you’re slacking off (in-person), they can tell you to focus”
Recommendations for the Program	“I’m not good at like focusing and stuff so like more hands-on stuff like being directly told what to do”
	“to have more interactive things as a group…… If people would communicate on their progress more”

## ABBREVIATIONS

C2: CURE Connections
CARE: Community Alliance for Research and Engagement
CHW: Community Health Worker
HSYACHW: High School and Young Adults Community Health Worker
MSM: Morehouse School of Medicine
NIH: National Institutes of Health
PGDx: Personal Genome Diagnostics
RE-AIM: (Reach, Effectiveness, Adoption, Implementation, Maintenance
STEM: Science, Technology, Engineering, Mathematics
THLP: Teen Health Leadership Program
UMB CURE: University of Maryland, Baltimore Continuing Umbrella of Research Experiences
UMSOM: University of Maryland School of Medicine
