# Supplementary material for: A Feasibility Study Describing the Successes and Challenges of Implementing a Virtual Community Health Worker Training Among High School Students Participating in a Summer STEM Enrichment Program
Source: J STEM Outreach. Author manuscript; Available in PMC 2024 Sep 26. (PMC11426545; doi:10.15695/jstem/v7i1.01)
Supplement: Virtual Community Health Worker Training Program – Vasanth Kumar, et al. Appendix. Interview Guide [file NIHMS2023832-supplement-Virtual_Community_Health_Worker_Training_Program___Vasanth_Kumar__et_al__Appendix__Interview_Guide.pdf]

JSO JOURNAL of STEM OUTREACH

Vol. 7, Issue 1, January 2024

Virtual Community Health Worker Training Program – Vasanth Kumar, et al.

Appendix. Interview Guide

Table A. *Final semi-structured interview guide questions.*

1. How was your experience with summer CURE program?
    a. What did you like about the CURE summer program? What didn't you like?
2. Were you able to finish all 18 Morehouse modules and receive the community health worker certificate?

    PROBES:
    *If "yes," what did they feel about the length of the modules? (skip to question 4)
    If "no" find out the reason.
3. Were you able to finish all the required Morehouse modules (modified) on the Canvas app?

    PROBES:
    *If "yes," ask if they will be willing to complete all module to get the CHW certificate.
    If "no," find out the reason.
    If "still working on it," ask if they look forward to completing the required modules and the extra module.
4. Can you tell us about how much time it took you to complete the modules?
    a. Were there modules that took you longer to complete?
5. What suggestions do you have to encourage the completion of all modules to get the CHW certification?
6. What are your thoughts about having CHW program as an in-person program as opposed to virtual program?
7. Do you have any recommendation to enhance the program experiences for the next year?
